# CCL2 and Lactate from Chemotherapeutics-Treated Fibroblasts Drive Malignant Traits by Metabolic Rewiring in Low-Migrating Breast Cancer Cell Lines

**DOI:** 10.3390/antiox13070801

**Published:** 2024-07-01

**Authors:** Maria Jesus Vera, Iván Ponce, Cristopher Almarza, Gonzalo Ramirez, Francisco Guajardo, Karen Dubois-Camacho, Nicolás Tobar, Félix A. Urra, Jorge Martinez

**Affiliations:** 1 Laboratory of Cellular Biology, Institute of Nutrition and Food Technology (INTA), University of Chile, Santiago 7830490, Chile; 2 Interdisciplinary Group on Mitochondrial Targeting and Bioenergetics (MIBI), Talca 3480094, Chile; 3 Laboratory of Metabolic Plasticity and Bioenergetics, Program of Molecular and Clinical Pharmacology, Institute of Biomedical Science (ICBM), Faculty of Medicine, University of Chile, Av. Independencia 1027, Santiago 7810000, Chile; 4 Network for Snake Venom Research and Drug Discovery, Santiago 7810000, Chile; 5 Interuniversity Center for Healthy Aging (CIES), Consortium of Universities of the State of Chile (CUECH), Santiago 8320216, Chile

**Keywords:** desmoplastic lesion, bioenergetics, mitochondrial respiration, immune response, antineoplastic drugs

## Abstract

While cytostatic chemotherapy targeting DNA is known to induce genotoxicity, leading to cell cycle arrest and cytokine secretion, the impact of these drugs on fibroblast–epithelial cancer cell communication and metabolism remains understudied. Our research focused on human breast fibroblast RMF-621 exposed to nonlethal concentrations of cisplatin and doxorubicin, revealing reduced proliferation, diminished basal and maximal mitochondrial respirations, heightened mitochondrial ROS and lactate production, and elevated MCT4 protein levels. Interestingly, RMF-621 cells enhanced glucose uptake, promoting lactate export. Breast cancer cells MCF-7 exposed to conditioned media (CM) from drug-treated stromal RMF-621 cells increased MCT1 protein levels, lactate-driven mitochondrial respiration, and a significantly high mitochondrial spare capacity for lactate. These changes occurred alongside altered mitochondrial respiration, mitochondrial membrane potential, and superoxide levels. Furthermore, CM with doxorubicin and cisplatin increased migratory capacity in MCF-7 cells, which was inhibited by MCT1 (BAY-8002), glutamate dehydrogenase (EGCG), mitochondrial pyruvate carrier (UK5099), and complex I (rotenone) inhibitors. A similar behavior was observed in T47-D and ZR-75-1 breast cancer cells. This suggests that CM induces metabolic rewiring involving elevated lactate uptake to sustain mitochondrial bioenergetics during migration. Treatment with the mitochondrial-targeting antioxidant mitoTEMPO in RMF-621 and the addition of an anti-CCL2 antibody in the CM prevented the promigratory MCF-7 phenotype. Similar effects were observed in THP1 monocyte cells, where CM increased monocyte recruitment. We propose that nonlethal concentrations of DNA-damaging drugs induce changes in the cellular environment favoring a promalignant state dependent on mitochondrial bioenergetics.

## 1. Introduction

Breast cancer (BC), a desmoplastic lesion, is mainly constituted by a stromal component of the tumor, which includes pericytes, immune cells, inflammatory cells, and fibroblasts (also known as cancer-associated fibroblasts or CAFs) [1]. As a central component of the tumor microenvironment (TME), the CAF population is highly heterogeneous in its functions, promoting extracellular matrix (ECM) remodeling, metabolic shifts, angiogenesis, and modulation of infiltrating immune cells [2,3]. This occurs by producing soluble factors such as growth factors, chemokines, cytokines, and exosomes [4].

Collaborative metabolic shifts between CAF and epithelial cancer cells have been extensively described in several cancers [5,6], promoting cancer cell survival and chemoresistance [7]. This fibroblast population exhibits high glucose uptake and secretes lactate to TME via monocarboxylate transporter 4 (MCT4), which in turn is incorporated via MCT1 and used as an energetic substrate by cancer cells [8,9]. In response to lactate availability, these cancer cells can increase mitochondrial mass and activity by SIRT1-dependent PGC-1a activation and promote mitochondrial transfer from CAFs [10]. Interestingly, lactate present in the conditioned medium from CAF promotes mitochondrial hyperpolarization, a decrease in Complex II and III levels, but an increase in Complex I. These alterations in the electron transport chain components are accompanied by the accumulation of succinate and fumarate without changes in α-ketoglutarate, suggesting a metabolic adaptation based on MCT1 activity [10]. It has been described that CAFs treated with chemotherapeutics increase cytokine expression and stimulate a high capacity for adhesion, invasiveness, and proliferation of highly metastatic BC cell lines [11]. On the other hand, low-migrating BC cells exhibit a promigratory phenotype and resistance to doxorubicin (Doxo), a DNA-damaging drug, in a medium with lower extracellular pH [12]. Despite the above, the metabolic adaptations in cancer cells driven by lactate and cytokines secreted from fibroblasts under DNA-damaging chemotherapeutics treatments still require more characterization.

Doxo and other chemotherapeutics targeting DNA function and structure are currently used as first-line chemotherapeutics in several cancer treatments [13]. These drugs initiate a cellular response directed at stopping replication and initiating DNA repair, known as DNA-damage response (DDR) [14], which is mediated by the activation of DNA-damage kinase sensors such as ATM [15]. In addition, DNA damage induces the production and secretion of bioactive factors [16]. Recently, it has been shown that conditioned medium from fibroblasts with oxidized ATM promotes increased migration of triple-negative BC cells without DDR but in a lactate-MCT4-MCT1 axis-dependent manner [17]. Among DNA-damage-related cytokines, the monocyte chemoattractant protein 1 (MCP1, also known as CCL2) has been reported as an active agent that modulates the migratory capacity of cells exposed to genomic damage [18,19]. To our knowledge, the effect of bioactive soluble factors secreted by fibroblasts under DNA-damaging drug treatment on mitochondrial metabolism and malignant traits in low-migrating BC cells remains elusive. 

In this work, we explore the effect of the conditioned medium from RMF-621 mammary fibroblasts treated with DNA-damaging drugs on mitochondrial bioenergetics and migration of low-migrating BC MCF-7, T47-D, and ZR-75-1 cell lines. For this purpose, RMF-621 cells were treated with cisplatin and Doxo, which bind to DNA structure and inhibit DNA replication and transcription [19,20], as well as BIBR-1532, a well-known telomerase inhibitor [20] at noncytotoxic concentrations. So far, most works related to chemotherapeutics drugs have been focused on DNA damage of the stromal component, which plays a crucial role in carcinogenesis, generating the effect of these compounds in epithelial carcinoma cells. Our results propose a drug-induced inflammatory pretumoral landscape that favors a metabolic remodeling dependent on lactate and glutamine utilization, which is required for stimulating epithelial motility.

## 2. Materials and Methods

### 2.1. Cell Culture, Cell Lines, and Chemicals

We used human cell line RMF-621, which corresponds to hTERT-immortalized mammary fibroblasts derived from a reduction mammoplasty obtained via a generous gift from Dr. Charlotte Kuperwasser (Tufts University, Boston, MA, USA) [21]. The RMF-621 cells were cultured in high-glucose (25 mM) Dulbecco’s modified Eagle’s medium (DMEM) (Invitrogen, Carlsbad, CA, USA), supplemented with antibiotics and 10% fetal bovine serum (FBS) (Hyclone, Logan, UT, USA) and maintained in a humidified atmosphere of 37 °C, 5% CO_2_. MCF-7, T47D, and ZR75-1; human epithelial breast adenocarcinoma cell lines were purchased from ATCC (Manassas, VA, USA), cultured in DMEM high glucose without pyruvate, supplemented with 10% FBS, and maintained under the same conditions as above. MCT1 inhibitor BAY-8002 was acquired from TOCRIS Bioscience (Minneapolis, MN, USA). Cisplatin, Doxorubicin, BIBR-1532, and antitubulin antibody were purchased in Sigma Aldrich (St. Louis, MO, USA). CCL2/MCP1 was from R&D systems (Minneapolis, MN, USA).

### 2.2. Preparation of Conditioned Media from Stromal Cells

RMF-621 cells (6.6 × 10^3^/cm^2^) were incubated in media enriched by 10% FBS for 72 h in the presence of BIBR-1532 (1.7 μM), Cisplatin (3 μM), and Doxorubicin (18 nM). After this, cells were incubated for 24 h in serum-free media, and the supernatant media were collected, centrifuged (8000× *g*, 5 min), and aliquoted for later use at −20 °C.

### 2.3. Lactate Quantification

Lactate was evaluated in conditioned media by RMF-621 cells (1 × 10^5^ cells in a 6-well plate) previously exposed to a 72-h treatment with 1.7 μM BIBR-1532, 3.0 μM Cisplatin, and 18 nM Doxorubicin. In the last 6 h of culture, the culture media was changed to phenol red-free media in the absence of serum, and lactate abundance in these media was measured with L-Lactate Assay kit of ScienceCell Laboratories (Carlsbad, CA, USA) according to the manufacturer’s instructions.

### 2.4. Mitochondrial ROS (mtROS) and Cytosolic ROS Levels

The mtROS levels were measured using staining with MitoSOX^®^ Red probe (Invitrogen, Carlsbad, CA, USA). Mammary fibroblasts (1.5 × 10^5^ cells/mL) were seeded into 6-well plates, incubated for 72 h with drugs, washed with PBS, and incubated with MitoSOX Red^®^ (5 µM) for 30 min. Then, they were recollected and washed, and the fluorescence was detected by flow cytometry. As a positive control, menadione (25 µM) was used.

Epithelial ZR-75, MCF-7, and T47D cancer cells (0.1 × 10⁶ cells/mL) were treated with conditioned medium diluted 1:1 with high-glucose DMEM medium for 24 h. After treatment, they were washed with PBS and incubated with MitoSOX^®^ Red (5 µM) or Dihydroethidium (5 µM, DHE, ThermoFisher Scientific, Waltham, MA, USA) for 30 min. Menadione (25 µM) was used as a positive control. Subsequently, cells were harvested and washed, and fluorescence was detected by FCS Calibur flow cytometry as described in [22].

### 2.5. MTT Assay

MTT assay was conducted to indirectly assess the viability of MCF-7 cells in conditioned media. Initially, 10,000 cells were seeded at 100 µL per well in 96-well microtiter plates and subsequently cultured in the conditioned media containing Control, BIBR-1532, Cisplatin, and Doxorubicin for 24 h. For treatment with menadione (0.5 μM), UK-5099 (10 μM), or EGCG (50 μM), it was introduced one h after the respective conditioned media had been applied. After 48 h of treatment, the MTT reduction was determined using a multireader Synergy H1 (Agilent, Santa Clara, CA, USA) as described [23].

### 2.6. Determination of Mitochondrial Membrane Potential (∆ψm)

MCF-7, T47D, and ZR75-1 cell lines (0.15 × 10^6^ cells/mL) were treated with conditioned media from stromal cells for 24 h. After treatment, cancer cells were washed with PBS and incubated with 5 nM tetramethylrhodamine methyl ester (TMRM, ThermoFisher Scientific, MA, USA) for 30 min as described at 37 °C [24]. FCCP (1 µM) was used as a positive control. Then, cells were washed and resuspended to detect the fluorescence using a BD FACSAria III flow cytometer.

### 2.7. Evaluation of Metabolism in Real-Time 

The real-time analysis of metabolism in RMF-621 and MCF-7 cells was performed using a Seahorse XFe96 Analyze RMF-621(Seahorse Agilent, Santa Clara, CA, USA). Cells were seeded (1 × 10^4^ cells/well) on XFe96 V3-PS multiwell plates and kept for 24 h at 37 °C in 5% CO_2_ with a DMEM high-glucose culture medium supplemented with 10% FBS. For RMF-621 cells, the culture medium was replaced with a fresh culture medium containing the DNA-damaging drugs until 72 h of exposition. For MCF-7 cells, the culture medium was replaced with CM from stromal cells until 24 h. To evaluate the mitochondrial respiration under standard culture conditions (with the full availability of glucose and glutamine), the culture medium of cells was replaced with glucose-based assay medium (unbuffered DMEM without red phenol, 4 mM glutamine, and 10 mM glucose, pH = 7.4) or lactate-based assay medium (unbuffered DMEM without red phenol, 4 mM glutamine, and 10 mM lactate, pH = 7.4) 1 h before the assay. Glucose- or lactate-dependent mitochondrial respiration was evaluated by the sequential injection of oligomycin (1 μM), FCCP (250 nM), and rotenone plus antimycin A (1 μM/1 μM) or two injections of FCCP (250 nM) and rotenone plus antimycin A (1 μM/1 μM), respectively. The glycolysis of MCF-7 cells was evaluated by injecting glucose (10 mM), oligomycin (1 µM), and 2-deoxyglucose (2-DG, 100 mM), according to Cordova-Delgado et al. [25]. The oxygen consumption rate (OCR) and extracellular acidification rate (ECAR) were determined by specific excitation and emission wavelengths of oxygen (532/650 nm) and protons (470/530 nm). Each experiment was performed at least in triplicate. OCR and ECAR data were normalized by protein content/well, which was determined using BCA kit.

### 2.8. Determination of Sub-G1 Population

RMF-621 cells were grown in 12-well plates and treated with DMSO, BiBr (1.7 µM), Cisplatin (Cis, 3.0 µM), and Doxorubicin (Doxo, 18 nM) for 48 h. Then, cells were permeabilized with methanol, treated with RNAse A for 1 h at 37 °C, collected, washed, and resuspended in a propidium iodide (PI) solution as described in [26]. The sub-G1 populations were analyzed by flow cytometry.

### 2.9. Cell Motility Assay

Low-migrating MCF-7, T47D, and ZR75-1 epithelial cell lines were used as a model of differential migration and invasion using a 6.5 mm Transwell chamber with a pore size of 8 µm (Corning, Steuben County, NY, USA). In these experiments, 5 × 10^4^ cancer cells were allowed to migrate for 24 h to the stimulus of 50% conditioned medium generated by RMF-621 cells previously cultured by 72 h in the presence of BIBR-1532, Cisplatin or Doxorubicin in the experimental conditions described above. To analyze the effect of stromal-derived lactate on epithelial migration and the role of MCT1 monocarboxylate transporters, a group of MCF-7 cells were stimulated to migrate under the stimulus of stromal conditioned media in the presence of BAY8002 (100 nM), an inhibitor of MCT1. After the migration period, the Transwell membrane was fixed in methanol, and migratory cells were stained on the lower side of the membrane with 0.2% crystal violet. The migration values correspond to the average of 3–4 independent experiments by counting 16 fields from 4 pictures (×20) per chamber (2 chambers per experimental condition). To evaluate the participation of mitochondrial metabolism in MCF-7, T47D, and ZR75-1 cell migration stimulated by CM from stromal cells, EGCG (50 mM), UK 5099 (10 mM), and rotenone (10 nM and 1 mM) were added 24 h before the assay and maintained during the 24 h time of migration assay.

### 2.10. Quantitative PCR

According to the manufacturer´s instructions, total RNA was isolated from stromal cells with Trizol (Ambion, Carlsbad, CA, USA). A reverse transcription to complementary DNA was performed with 1 mg of RNA from each sample using an M-MLV reverse transcriptase and oligo-dT (Promega, Madison, WI, USA) as a primer, according to the manufacturer’s protocol. Glut1 and MCT4 messenger RNA (mRNA) expression was assessed via real-time PCR using a LightCycler instrument (Roche, Basel, Switzerland). The reaction was performed using 200 ng of complementary DNA and a LightCycler1 FastStart DNA Master SYBR Green I kit (Roche) with a final volume of 10 mL. All the reactions were performed in duplicate, and negative controls were included. GAPDH was used as housekeeping. The primer sequences are described in Table 1.

### 2.11. Western Blot and Antibodies

The MCT1 protein levels were evaluated by Western blot. Briefly, cells previously plated for 72 h with drugs were lysed in lysis buffer (30 mM Tris-HCl pH 7.5, 5.0 mM EDTA, 150 mM NaCl, 1% Triton X-100, 0.5% sodium deoxycholate, 0.1% SDS, and 10% glycerol) supplemented with complete protease inhibitors (Roche, Mannheim, Germany). Pellets were incubated for 1 h in lysis buffer at 4 °C and then centrifuged at 14,000× *g* for 15 min at 4 °C, keeping the supernatants. The protein concentration of cell lysates was determined using a Pierce BCA Protein Assay kit (Thermo, Rockford, IL, USA). Protein extracts were denatured in sodium dodecyl sulfate (SDS)–polyacrylamide gel electrophoresis loading buffer 4 (240 mM Tris–HCl, pH 6.8, 8% SDS, 40% glycerol, and 20% 2-mercaptoethanol), incubating the samples for 1 h at 37 °C. Equal amounts of protein from different treatments were resolved by SDS–polyacrylamide gel electrophoresis in 10% acrylamide gels and electrotransferred to polyvinylidene difluoride membranes using a buffer containing 24 mM Tris, 194 mM glycine, and 20% methanol. The proteins were further analyzed using the Supersignal West Dura Extended Duration Substrate (Thermo, Rockford, IL, USA). Immunoreactions were achieved by incubation of the membranes, previously blocked with a solution containing 5% bovine serum albumin in Tris-buffered saline and 0.05% Tween 20 (Sigma, St. Louis, MO, USA), with anti-MCT1 (Santa Cruz, CA, USA), and mouse anti-alpha tubulin (#T5168) from Sigma (St. Louis, MO, USA). Densitometric analysis of Western blot bands was performed using a C-Digit Blot Scanner and Image Studio Digits software v.5.2 from LI-COR Biosciences (Lincoln, NE, USA). The uncropped gels are shown in the Supplementary Materials File.

### 2.12. Generation of THP-1 Macrophages with M1 Profile

Human THP-1 cell line (ATCC^®^ TIB-202™) was maintained in RPMI 1640 (Corning) supplemented with 10% fetal bovine serum (FBS) (Life Technologies, Carlsbad, CA, USA), 5 µM β-mercaptoethanol (Sigma), 100 U/mL penicillin, and 100 µg/mL streptomycin incubated at 37 °C and 5% CO_2_. A 72 h protocol was used for macrophage differentiation as previously described [27]. Briefly, cells were treated with 10 ng/mL 13-phorbol 12-myristate acetate (PMA) (Sigma) for 48 h, followed by 24 h without the stimuli. M1 proinflammatory macrophages were differentiated by LPS (0.1 ng/mL) and IFNg (20 ng/mL) stimuli. Conditioned medium from RFM cells (prestimulated with BiBr, Doxo, Cis, and Vehicle) was used as costimulus with LPS + IFNg for 24 h. The CD40, CD80, CD163, and CD207 membrane markers were measured by flow cytometry (FACS Canto BD, Piscataway, NJ, USA).

### 2.13. Statistics

The data are expressed as mean ± standard deviation (SD) of three to four independent experiments. Statistical analysis was performed using one-way or two-way ANOVA with Bonferroni’s post-test for pairwise comparisons with Graph Pad Prism 4.03 (GraphPad Software, San Diego, CA, USA). The data were considered statistically significant when *p* < 0.05.

## 3. Results

### 3.1. Drugs Affecting the Structure and Function of DNA Impact the Mitochondrial Bioenergetics in Mammary Stromal Cells

**Figure 1:**
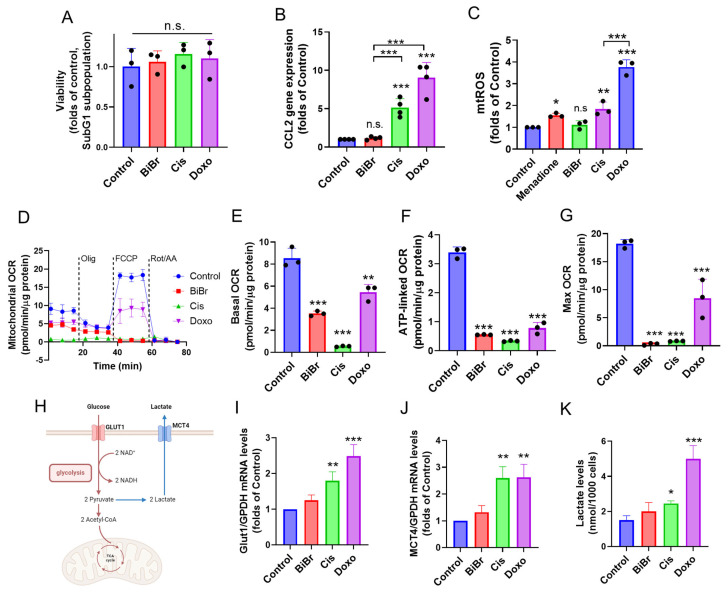
DNA-damaging chemotherapeutics promote metabolic remodeling in fibroblast RMF-621 cells: (**A**) effect of BiBr (1.7 µM), Cisplatin (Cis, 3.0 µM), and Doxorubicin (Doxo, 18 nM) on sub-G1 population at 72 h of treatment, (**B**) relative *CCL2* gene expression measured by qPCR, (**C**) mitochondrial ROS levels (mtROS) measured using mitoSOX dye by flow cytometry, (**D**) oxygen consumption rate (OCR) profile, (**E**–**G**) changes in basal, ATP-driven, and maximal respirations of RMF-621 cells treated with BiBr, Cis, and Doxo for 48 h, (**H**) schematic diagram on lactate production, (**I**,**J**) changes in *Glut1* and *MCT4* gene expression in RMF-621 cells treated with BiBr (1.7 μM), Cisplatin (3 μM, Cis), and Doxorubicin (18 nM, Doxo) for 72 h, (**K**) lactate levels in the media of RMF-621 after treatment with DNA-damaging drugs. Data are shown as the mean ± SD of three or four independent experiments. * *p* < 0.05, ** *p* < 0.01, *** *p* < 0.001, vs. control (DMSO). n.s.: not significant.

To analyze whether drugs that affect DNA structure can modify the cellular metabolic behavior, we subject mammary stromal cells (RMF-621 cells) to a 72 h treatment with increased concentrations of BIBR-1532 (BiBr), a well-known telomerase inhibitor [20], Cisplatin (Cis), a drug that after binding to DNA, interferes with normal transcription [28], and Doxorubicin (Doxo), a drug able to intercalate with DNA base pairs and is commonly used in breast cancer therapy [29]. As Supplementary Figure S1 shows, 1.7 μM BIBR, 3.0 μM Cis, and 18 nM Doxo inhibit the MTT reduction (Supplementary Figure S1A) and decrease the total cell number without increasing the number of Trypan blue positive cells (Supplementary Figure S1B), suggesting that these selected concentrations mainly produce a reduction in proliferation. To confirm the absence of cytotoxicity, the effect of these DNA-damaging drugs on the sub-G1 subpopulation, which represents apoptotic cells, was evaluated at 72 h of treatment. No significant changes were observed in the sub-G1 subpopulation (Figure 1A).

In these noncytotoxic concentrations, either Cis or Doxo stimulated the expression of *CCL2*, a specific factor involved in stromal–cancer cell communication (Figure 1B). Notably, RMF-621 treated with Cis as well as Doxo increased the mitochondrial ROS (mtROS) production at 72 h of treatment (Figure 1C) and exhibited changes in the oxygen consumption rate (OCR) profile (Figure 1D), which was characterized by reduced basal (Figure 1E), ATP-linked (Figure 1F), and maximal respiration (Figure 1G). These results suggest that these drugs produce a nonproliferative phenotype that involves a reduction in mitochondrial bioenergetics.

### 3.2. Drugs Affecting the Structure and Function of DNA Induce Glucose Reprogramming in Mammary Stromal Cells

Since DNA-damaging drugs reduced the mitochondrial respiration in RMF-621 cells, we evaluate a possible metabolic remodeling toward glycolysis, increasing lactate production (Figure 1H). As Figure 1I,J shows, Cis and Doxo increased the mRNA levels of Glut1, responsible for glucose uptake (Figure 1I), and monocarboxylate transporter MCT4, which is involved in the release of lactate [30] (Figure 1J). After a 72-h treatment, either Cis or Doxo also significantly stimulated the lactate production (Figure 1K), suggesting that both drugs stimulate a metabolic flux involving glucose uptake and lactate export in RMF-621 stromal cells. To determine if the genomic action of these drugs, which activate the DNA-damage kinase sensor ATM, affected some determinant of glucose reprogramming (as MCT4), we added 5 μM KU55933 into the drug-contained culture media that stimulate MCT4. Supplementary Figure S1C shows that the ATM inhibitor blocked the increase in MCT4 levels induced by the three drugs under study. This suggests that DNA damage caused by these drugs may mediate the metabolic remodeling toward a lactate secretor phenotype.

### 3.3. Conditioned Media from Stromal Cells Exposed to DNA-Damaging Drugs Increases the Migration in Epithelial Breast Cancer Cells

**Figure 2:**
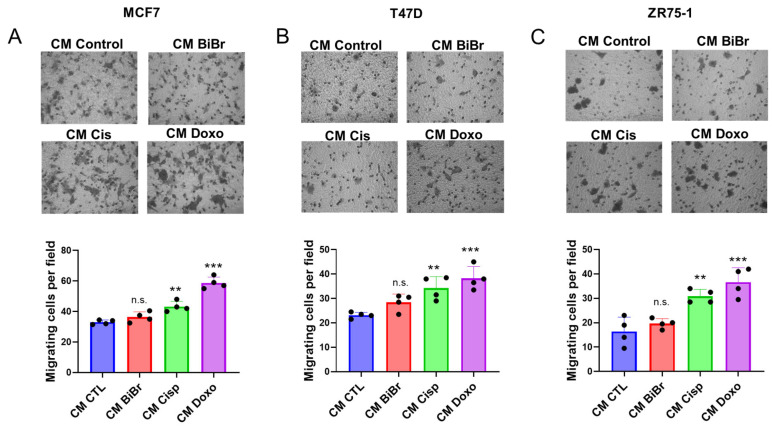
Conditioned media from DNA-damaging drugs increase motility in low-migrating epithelial breast cancer cells: (**A**–**C**) effect of CM on the migration of MCF-7, T47D, and ZR75-1 breast cancer cell lines. Data are shown as the mean ± SD of four independent experiments. ** *p* < 0.01, *** *p* < 0.001, vs. control (DMSO). n.s.: not significant.

To assess whether soluble factors generated by drug-treated RMF-621 cells affect the cell motility of epithelial cells, we performed a migratory assay (described in Material and Methods) with three epithelial breast cancer cell lines. The main property that shares these cell lines is their low migrating potential. As Figure 2 shows, the three cell lines exposed during the migration assay to media conditioned by Cis- and Doxo-treated RMF-621 cells migrate following a very similar pattern, being media conditioned in the presence of Doxo, which exerts the more potent migratory stimulus. These results suggest that DNA-damaging drugs induce the secretion of soluble factors in RMF-621, which triggers changes in the migration capacity of low-migrating breast cancer cell lines.

### 3.4. Conditioned Media from Stromal Cells Exposed to DNA-Damaging Drugs Increases the Metabolic Plasticity of Epithelial Breast Cancer Cells

**Figure 3:**
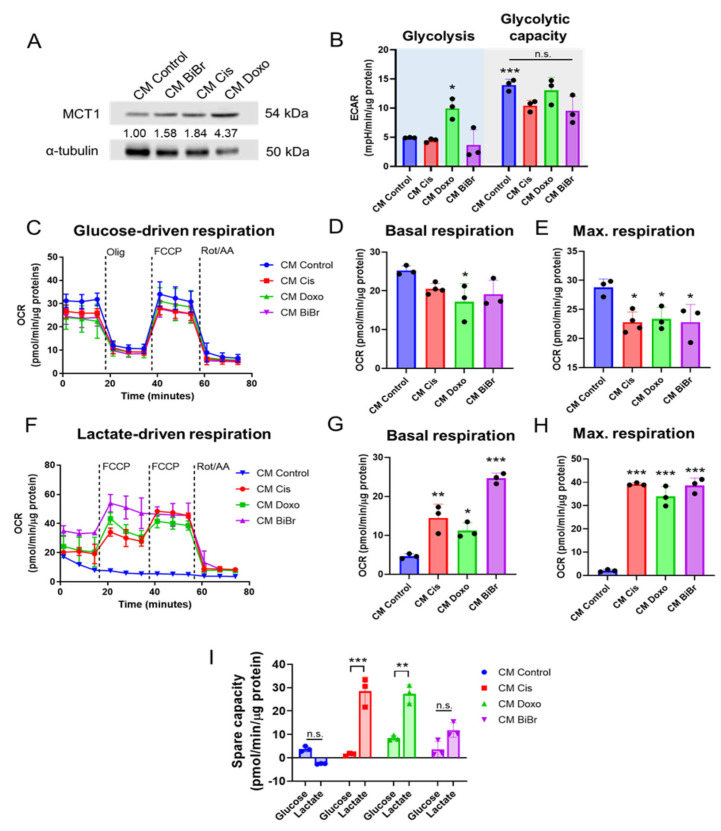
Conditioned media from DNA-damaging drugs increase metabolic plasticity dependent on lactate/MCT1 in MCF-7 cells: (**A**,**B**) effect of CM on MCT1 levels and glycolysis and glycolytic capacity in MCF-7 breast cancer cells at 72 h of exposition, (**C**–**E**) effect of CM on the profile of respiration, basal, and maximal respirations of MCF-7 exposed to Seahorse assay buffer containing 10 mM glucose and 4 mM glutamine, (**F**–**H**) effect of CM on the profile of respiration, basal, and maximal respirations of MCF-7 exposed to Seahorse assay buffer containing 10 mM lactate and 4 mM glutamine, (**I**) differences in the spare capacity dependent on glucose or lactate presence in MCF-7 cells exposed to CM by DNA-damaging drugs for 24 h. Data are shown as the mean ± SD, N = 4 independent experiments. * *p* < 0.05, ** *p* < 0.01, *** *p* < 0.001, vs. control (DMSO). n.s.: not significant.

Based on previous results, we speculate that the stromal soluble factors secreted by RFM-621 exposed to the DNA-damaging drugs could supply lactate as an alternative energetic substrate for the metabolism of epithelial breast cancer cells. To explore this, we selected the MCF-7 cell line for metabolic analysis. As Figure 3A shows, Cis and Doxo increased the MCT1 protein levels, the monocarboxylate transporter involved in cellular lactate uptake, in MCF-7 cells treated for 72 h with the conditioned media (CM) from drug-treated stromal cells. Under these conditions, glycolysis was only increased by CM from stromal cells treated with Doxo (Figure 3B, *p* < 0.05 vs. CM Control), and the glycolytic capacity, a parameter that reports the adaptive response to mitochondrial stress [31], was unaltered (Figure 3B). Consistent with this, only CM Doxo produced a significant reduction in basal respiration (Figure 3C,D), and CM from three antineoplastic drugs reduced the maximal mitochondrial respiration (Figure 3E) without effects on ATP-linked respiration (Supplementary Figure S2) when exposed to glucose (10 mM) and glutamine (4 mM), suggesting a reduction in the mitochondrial substrate oxidation. Then, we evaluate whether MCF-7 cells treated with CM for 24 h and exposed to lactate for 1 h could adapt the substrate preference to maintain mitochondrial respiration. Our results show that basal and maximal respirations (under two FCCP injections) were increased when MCF-7 cells were exposed to CM from antineoplastic drugs (Figure 3F–H). Consistent with the suppressive effect of mitochondrial respiration by exogenous lactate treatment in some cancer cell lines [32], they failed to support mitochondrial respiration when MCF-7 cells were treated with CM Control. In contrast, CM Cis and CM Doxo increased spare capacity (which reports the difference between maximal OCR and basal OCR) in MCF-7 cells when lactate (10 mM) supported respiration (Figure 3I). Together, these results suggest that lactate and other soluble factors in the CM Cis and Doxo trigger a metabolic adaptation with increased ability for lactate-dependent mitochondrial respiration and spare capacity in MCF-7 epithelial breast cancer cells.

### 3.5. CCL2 and Lactate from Conditioned Media by DNA-Damaging Drugs Are Essential for Inducing an Increased Migratory Capacity of MCF-7 Cancer Cells

**Figure 4:**
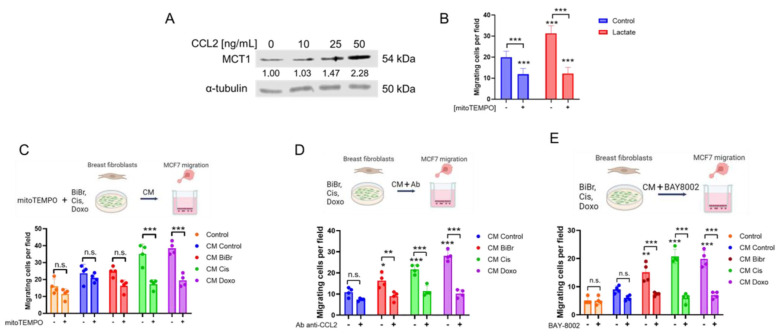
CCL2 and lactate from conditioned media by DNA-damaging drugs are essential for increased migration in MCF-7 cancer cells: (**A**) effect of increasing concentrations of CCL2 on MCT1 protein levels in MCF-7 cells at 72 h of exposition, (**B**) effect of lactate (20 mM) on migration of MCF-7 cells at 24 h of exposition, (**C**) effect of stromal CM prepared by DNA-damaging drugs (as explained above) and with a previous incubation (1 h) of mitoTEMPO (1 µM) on MCF-7 migration, (**D**,**E**) effect of CM produced by DNA-damaging drugs and then the addition of blocking antibody anti-CCL2 (10 µg/mL) or MCT1 inhibitor, BAY-8002 (100 nM) on MCF-7 migration. Data are shown as the mean ± SD, N = 4 independent experiments. * *p* < 0.05, ** *p* < 0.01, *** *p* < 0.001, vs. control (DMSO). n.s.: not significant.

Based on our previous results, we speculate that the increased *CCL2* gene expression in RMF-621 stromal cells involved the increased secretion and presence of CCL2 in the CM, which could trigger the increase in MCT1 protein levels in MCF-7 epithelial cells (Figure 3A). We observed that treatment with increased CCL2 concentrations correlates with enhanced MCT1 protein levels in MCF-7 (Figure 4A), and 20 mM lactate in the culture medium stimulates MCF-7 migration, a phenomenon that is blocked by the addition of mitoTEMPO (Figure 4B), a well-known mitochondrial antioxidant [33].

We perform migration assays under different conditions to determine whether cell migration, an essential feature of epithelial malignancy, is affected by soluble factors such as CCL2 and lactate produced by drug-treated stromal cells. As Figure 4C shows, MCF-7 cell migration is significantly stimulated by drug-treated media-conditioned RMF-621, which was not observed in CM from stromal cells previously treated with mitoTEMPO (Figure 4C), suggesting that an increased oxidative environment is required for the constitution of the soluble factors in the CM, as observed in Figure 1E. Taking into account that RMF-621 cells treated with DNA-damaging drugs exhibited increased CCL2 gene expression, lactate, and mtROS production, and the possibility that this factor activates cell migration, we suppress the drug-treated stromal migration stimulus including a specific anti-CCL2 blocking antibody and, alternatively, 100 nM BAY-8002, a specific inhibitor of MCT1 (that allows the uptake of lactate in epithelia) [34], into the migration assays. Notably, the increased MCF-7 migration stimulated by CM from stromal cells was abolished (Figure 4D,E). These results suggest that the CCL2/MCT1/lactate axis could be relevant in the malignant features of MCF-7 cells exposed to CM from stromal cells treated with DNA-damaging drugs.

### 3.6. Mitochondrial Pyruvate Transport and Glutaminolysis Are Required for Maintaining the Viability and Migration of Epithelial Breast Cancer Cells Exposed to CM from DNA-Damaging Drugs

**Figure 5:**
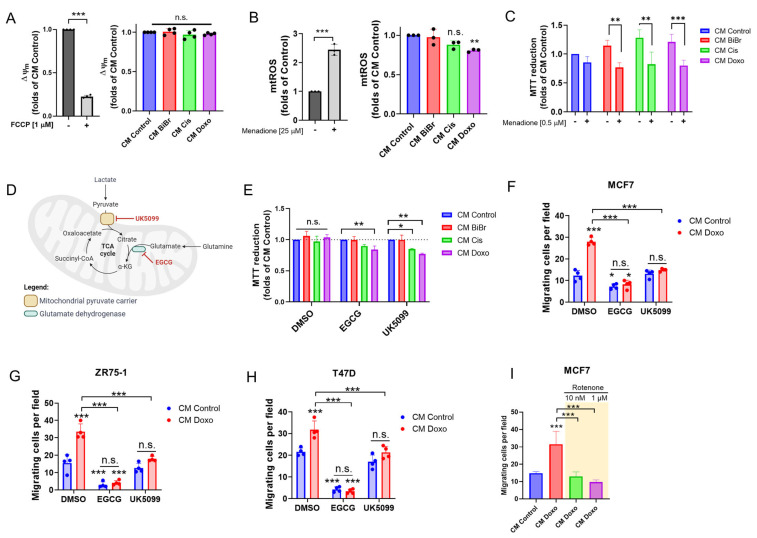
Mitochondrial pyruvate transport and glutaminolysis are required for maintaining the viability and migration of breast cancer cells exposed to CM from DNA-damaging drugs: (**A**) mitochondrial membrane potential (Δψm), (**B**) and mitochondrial superoxide production in MCF-7 cells exposed to CM from stromal cells for 24 h. FCCP (1 µM) and menadione (25 µM) were used as positive controls. (**C**) Vulnerability of MCF-7 cells exposed to CM from stromal cells for 48 h in the presence of a low concentration of menadione (0.5 µM), (**D**) diagram of mitochondrial utilization of energy substrates as pyruvate (which can be derived from glucose or lactate and incorporated into mitochondrion by mitochondrial pyruvate carrier, MPC, in IMM) and glutaminolysis, showing two inhibitors used in this study, (**E**) effect of MPC (5 µM UK5099) and glutamate dehydrogenase (25 µM EGCG) inhibitors on viability of MCF-7 cells exposed to CM from stromal cells treated with DNA-damaging drugs for 48 h, (**F**–**H**) effect of 25 µM EGCG and 5 µM UK5099 on the MCF-7, ZR75-1, and T47D cell migration stimulated by CM from stromal cells treated with doxorubicin (18 nM Doxo), (**I**) effect of rotenone (10 nM and 1 µM Rot, Complex I inhibitor) on MCF-7 migration. Data are shown as the mean ± SD, N = 4 independent experiments. * *p* < 0.05, ** *p* < 0.01, *** *p* < 0.001, vs. control (DMSO). n.s.: not significant.

Since CM from stromal cells promoted an increased ability for lactate-dependent mitochondrial respiration in MCF-7 cells, we evaluate whether this affects the mitochondrial membrane potential and ROS levels in MCF-7, T47D, and ZR75-1 cells. There were no changes in Δψm, but a total ROS and mitoROS (for MCF-7 cells) reduction was observed in breast cancer cells treated with CM Cis and Doxo from stromal cells (Figure 5A,B and Figure S3). Notably, these MCF-7 cells with reduced mtROS were significantly vulnerable to menadione, a pro-oxidant small molecule that inhibits the mitochondrial electron transport chain (ETC) [35,36,37]. At the same time, no changes were exhibited for MCF-7 treated with CM control (Figure 5C). This suggests that substrate preference for lactate could be related to increased antioxidant defenses [38] and elevated ETC activity.

It has been reported that lactate uptake promotes glutaminolysis, supporting oxidative metabolism in cancer cells in an MCT1-dependent manner [39,40]. Based on this evidence, we evaluate if lactate-derived pyruvate (produced by lactate dehydrogenase A, LDHA), which could be incorporated into mitochondria by mitochondrial pyruvate carrier (MPC), and mitochondrial glutamine metabolization by glutamate dehydrogenase (GDH) contribute to filling the tricarboxylic acid (TCA) cycle during viability and enhanced breast cancer migratory ability (Figure 5D). As Figure 5E shows, the viability of MCF-7 cells was reduced in both CM Cis and Doxo in the presence of MPC inhibitor UK5099. In addition, only CM Doxo-treated MCF-7 cells in the presence of GDH inhibitor EGCG reduced the viability. On the other hand, we evaluated the effect of inhibitors of MPC, GDH, and respiratory complex I (rotenone) on the migration of breast cancer cell lines (MCF-7, T47D, and ZR75-1) stimulated by CM Doxo, showing complete inhibition (Figure 5F–I). These results suggest that CCL2/lactate-containing CM from stromal cells promotes mitochondrial pyruvate transport and glutaminolysis, supporting the enhanced migratory ability of epithelial breast cancer cells.

### 3.7. Soluble Factors Generated by Stromal Cells Exposed to Drugs Enhance THP-1 Recruitment in a mtROS and CCL2 Production-Dependent Manner

**Figure 6:**
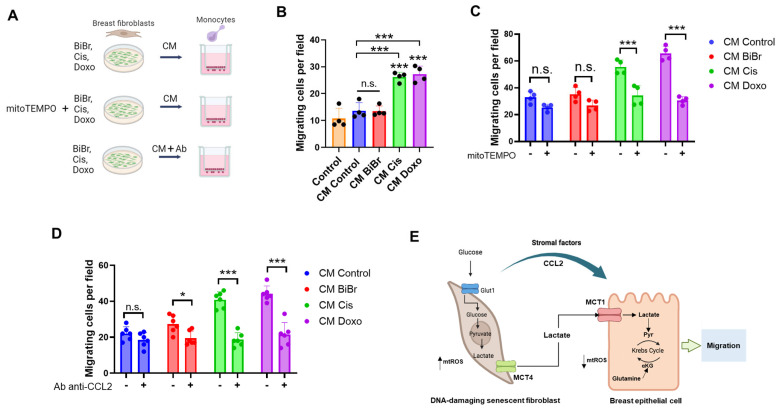
DNA-damaging drugs promote enhanced monocyte THP-1 recruitment: (**A**) diagram of experimental evaluation of conditioned media (CM) from fibroblasts RMF-621 treated with DNA-damaging drugs on migration, (**B**) effect of CM on THP-1 migration, (**C**) effect of CM produced by previous incubation (1 h) of mitoTEMPO (1 µM) and then DNA-damaging drugs on THP-1 migration, (**D**) effect of CM produced by DNA-damaging drugs and then the addition of blocking antibody anti-CCL2 (10 µg/mL) on THP-1 migration, (**E**) working model proposed in this work. Data are shown as the mean ± SD, N = 4–6 independent experiments. * *p* < 0.05, *** *p* < 0.001, vs. control (DMSO). n.s.: not significant.

Epidemiological studies have shown that chronic inflammation predisposes people to acquire various types of cancer [41,42], and depending on the microenvironmental cues in the tumor, macrophages switch metabolism and function according to the niche-derived stimuli sensed [42]. To analyze whether exposure to DNA-damaging drugs promotes an inflammatory environment that favors tumor development, we investigate whether Cis and Doxo regulate the infiltration of migratory cells for maintaining an inflammatory microenvironment. For this, we evaluate the capacity of media conditioned by RMF-621 cells treated with DNA-damaging drugs to recruit a human THP-1 monocyte cell line (Figure 6B). Based on the observation that RMF-621 cells treated with Cis and Doxo exhibited increased mtROS (Figure 1), we prepared stromal conditioned media with these antineoplastic drugs in the presence or absence of 1 µM mitoTEMPO [33], and the THP-1 migration was determined. As Figure 6C shows, stromal RMF-621 cells incubated for 72 h with mitoTEMPO cannot stimulate monocyte recruitment. Using an MCP-1/CCL2 blocking antibody in the migratory assay, we found that the blockade by the antibody partially annulled the recruitment in all cases, including the control condition (Figure 6D), suggesting a definitive role for MCP-1/CCL2 in the establishment of a protumoral inflammatory environment. On the other hand, we evaluated the effect of CM from antineoplastic drug-treated RMF-621 cells on metabolism and immunophenotype markers for M1 macrophages. Our results show that CM from three drugs significantly reduced the mitochondrial ROS production compared with CM control, without affecting the mitochondrial membrane potential. Moreover, Doxo increased CD80 and CD86, and Cis increased CD40 markers (Supplementary Figure S4).

## 4. Discussion

BC cells and their TME establish metabolic reciprocal interactions that contribute to the emergence of a more aggressive state [43]. Paradoxically, the present study highlights the consequences of noncytotoxic concentrations of DNA-damaging agents with clinical antineoplastic uses on mammary stromal cell metabolism and communication with epithelial cancer cells to support malignant features dependent on mitochondrial bioenergetics.

Under an epithelial–stromal metabolic coupling, stromal fibroblasts behave as a catabolic phenotype producing lactate [6]; meanwhile, carcinoma cells assume an anabolic role by consuming lactate that promotes cell proliferation and growth [44]. Thus, this metabolic arrangement would predominantly achieve biomass accumulation, which is more than an energetic task [45], and its implementation is of crucial importance in cells subjected to genomic damage. Our results show that chemotherapeutics (such as Doxo and Cis) that interfere with DNA structure and function promote a metabolic remodeling in breast fibroblasts, involving high mtROS production, reduced mitochondrial oxygen consumption, and increased lactate release mediated by MCT4, which is preferentially expressed in highly glycolytic tissues [23].

DNA damage is characterized by the production of several soluble factors [46,47], such as the chemokine (CC-motif) Ligand 2 (CCL2), which can actively recruit macrophages and T cells to the sites of genomic injury [38]. Although it has been described that CCL2 modulates the metabolism and proliferation of BC cells [18], the effect of CCL2 on the preference for energy substrates such as lactate and glutamine has yet to be completely understood. Our results suggest that CCL2 secretion from chemotherapeutics-induced fibroblasts increases MCT1 levels and, consequently, produces a high spare respiratory capacity for lactate oxidation without alterations in Δψm and mtROS levels in MCF-7 cells. Interestingly, these cancer cells showed increased migration and metabolic rewiring. Consistent with reports that show lactate uptake promotes glutaminolysis for supporting oxidative metabolism in cancer cells in an MCT1-dependent manner [30,31], we observed (in three different cell lines) that increased migration is dependent on MCT1, glutamate dehydrogenase, mitochondrial pyruvate carrier, and respiratory complex I, suggesting that lactate-derived pyruvate and glutamine-derived αKG oxidations are required for mitochondrial bioenergetics during migration stimulated by CM Cis and Doxo.

We also observed increased mtROS production in chemotherapeutics-induced fibroblasts, which was involved in the promigratory stimuli; however, both breast cancer cells and THP-1 cells exhibited a partial reduction in mtROS levels. While RMF-621 cells treated with mitochondrial-targeted antioxidant mitoTEMPO lacked the promigratory stimuli on MCF-7 and THP-1 cells, a low-concentration of menadione (a mitochondrial pro-oxidant and ETC inhibitor) triggered viability reduction in MCF-7 cells grown in CM from fibroblasts treated with DNA-damaging drugs. These cell-type-specific differences in the lactate-dependent redox thresholds may be related to lactate accumulation that can increase antioxidant defenses [38] and offer potential strategies for disrupting stromal-cancer epithelial communication.

Although our study does not identify a signaling pathway involved in the lactate-dependent metabolic rewiring of epithelial cells, its intracellular accumulation may have both cellular signaling and energy substrate roles [48]. It was recently described that intracellular lactate accumulation regulates energy metabolism by epigenetic modulation mediated by lysine lactylation (Kla) of histone promoters of *HK-1* and *IDH3G* [49], genes that encode hexokinase, the first enzyme in the glycolysis reactions, and isocitrate dehydrogenase 3 that catalyzes the formation of αKG and NADH in TCA cycle, respectively [50]. In BC patient samples with poor prognosis, *NDUFAF6*, a Kla-specific gene that is involved in the Complex I assembly and activity, is overexpressed [51]. Furthermore, LDHD, one of the five isoforms of lactate dehydrogenase [52], was identified to oxidize lactate and add electrons to the mitochondrial ETC at the level of cytochrome c, promoting lactate-dependent respiration [53]. This emerging evidence suggests complex roles for lactate in cancer cells, and if it occurs during metabolic coupling driven by chemotherapeutics-induced fibroblast, further studies will be required. Additionally, during the last decades, a significant effort has been made to block lactate transport and, in this manner, to avoid the building of a functional tumoral metabolism [54]. One target for this action has been the monocarboxylate transporters, whose inhibition with small molecules has reported interesting results in the clinical field [55,56].

CCL2 is the main chemokine and activator of monocytes, macrophages, and neutrophils [57], and it plays a crucial role in amplifying the inflammatory cascade related to chronic inflammation [58]. Previously, we demonstrated that the TNFα-dependent CCL2 secretion by mammary stromal cells depended on an oxidative environment, promoting monocyte and epithelial migration [59]. Our present results add a relevant action of CCL2 on lactate transporter protein levels and, therefore, changes in mitochondrial substrate preferences in epithelial cancer cells. Overall, from these data, CCL2 appears as a critical mediator in the metabolic response of cells to DNA insults.

Protumoral niche refers to microenvironmental conditions predisposing to cancer development [42,60], which may be influenced by inflammation [61]. Our results show that noncytotoxic concentrations of Cis and Doxo provoke an inflammatory response in mammary stromal cells, which is expressed in a higher expression of inflammatory marker (CCL2), mtROS, and monocyte recruitment. These results are mutually associated since CCL2 present in media conditioned by stromal cells appears to be the main one responsible for monocyte recruitment. More specifically, it seems that an inflammatory (and oxidative) environment constitutes a requisite for CCL2 in human monocytic THP-1 cells, as other authors have suggested previously [62]. Taken together, the singularity of our study lies in two fundamental facts: (i) it constitutes an effort to incorporate the most abundant tumoral phenotype (the stromal fibroblasts) into the tumor metabolic regulation, and (ii) it incorporates the concept that a noncytotoxic concentration of DNA-damaging agents can affect the inductive stromal behavior that, gradually, can induce epithelial malignancy. Further studies considering these two aspects can provide the basis for designing specific anticancer tools.

## 5. Conclusions

Our results encourage us to propose that DNA-damaging chemotherapeutics with clinical uses may change the stroma phenotype and paradoxically be a cause rather than just the consequence of the malignant process based on lactate- and glutamine-dependent metabolic rewiring, which may trigger therapeutic failure and resistance [63]. Since the stromal–epithelial metabolic communication and establishment of a mtROS-dependent inflammatory state favor the epithelial malignant propensity [8,41], potential redox-threshold-modifying agents may differentially offer new anticancer therapeutic strategies.

## Figures and Tables

**Table 1:** Primer list used for qPCR.

Accession Number	Target mRNA	Forward Primer (5′_3′)	Reverse Primer (5′_3′)
NM_002982.4	*CCL2*	TGTCCCAAAGAAGCTGTGATCT	GGAATCCTGAACCCACTTCTG
NM_006516.4	*Glut1*	CCAGCTGCCATTGCCGTT	GACGTAGGGACCACACAGTTGC
NM_047437037.1	*MCT4*	ATTGGCCTGGTGCTGCTGATG	CGAGTCTGCAGGAGGCTTGTG
NM_002046.7	*GAPDH*	TTGCCATCAATGACCCCTTC	TGATGACAAGCTTCCCGTTC

## Data Availability

Dataset available on request from the authors.

## Supplementary Materials

The following supporting information can be downloaded at https://www.mdpi.com/article/10.3390/antiox13070801/s1, Figure S1: Effect of DNA-damaging drugs on MTT reduction, cell number, and MCT4 levels. Figure S2: Effect of CM from RMF-621 treated with DNA-damaging drugs on ATP-linked respiration of MCF-7 cells. Figure S3: Effect of CM from RMF-621 treated with DNA-damaging drugs on mitochondrial membrane potential (Δψm) and ROS levels in low-migrating breast cancer cells. Figure S4: Effect of CM from DNA-damaging senescent fibroblasts on bioenergetics and inflammatory membrane markers in THP1-macrophages.
