# Supplementary material for: CCL2 and Lactate from Chemotherapeutics-Treated Fibroblasts Drive Malignant Traits by Metabolic Rewiring in Low-Migrating Breast Cancer Cell Lines
Source: Antioxidants (Basel). 2024 Jul 1;13(7):801. doi: 10.3390/antiox13070801 (PMC11274190; doi:10.3390/antiox13070801)
Supplement: Supplementary file 1 [file antioxidants-13-00801-s001.zip › antioxidants-3023630-supplementary.pdf]

CCL2 and Lactate from Chemotherapeutics-Treated Fibroblasts Drive Malignant Traits by Metabolic Rewiring in Low-Migrating Breast Cancer Cell Lines

Maria Jesus Vera, Iván Ponce, Cristopher Almarza, Gonzalo Ramirez, Francisco Guajardo, Karen Dubois-Camacho, Nicolás Tobar, Félix A. Urra, Jorge Martinez.

SUPPLEMENTARY INFORMATION

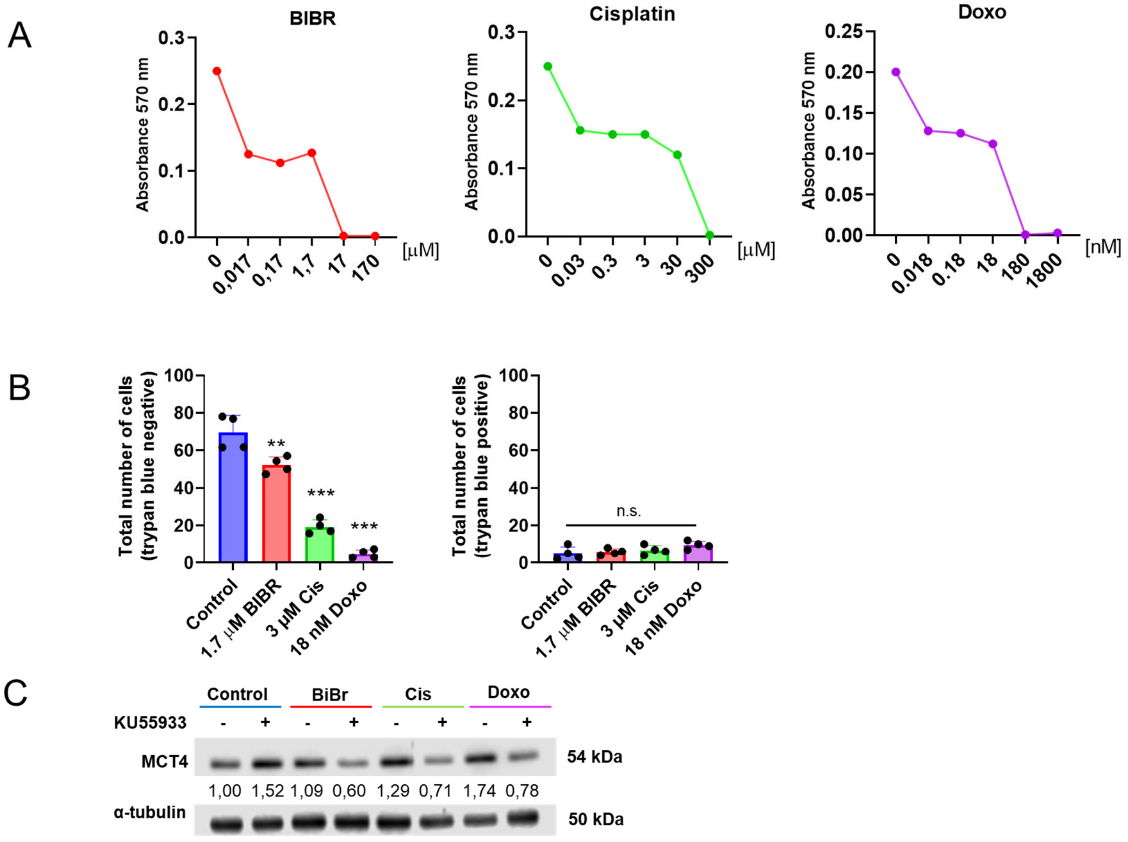

Supplementary Figure S1: Effect of DNA-damaging drugs on MTT reduction, cell number, and MCT4 levels. **A.** Effects of increased concentrations of BIBR, cisplatin, and doxorubicin on MTT reduction of RMF-621 cells at 72 h of treatment. **B.** Effect of selected non-cytotoxic concentrations on total numbers of RMF-621 cells exposed at 72 h of treatment. The number of cells was determined by the Trypan blue exclusion method. **C.** Effect of ATM inhibitor, KU55933 (5 µM), on increased MCT4 protein levels induced by DNA-damaging drugs. Data are shown as the mean ± SD of three independent experiments. **p* < 0.05, ***p* < 0.01, ****p* < 0.001, vs. Control (DMSO). n.s.: not significant.

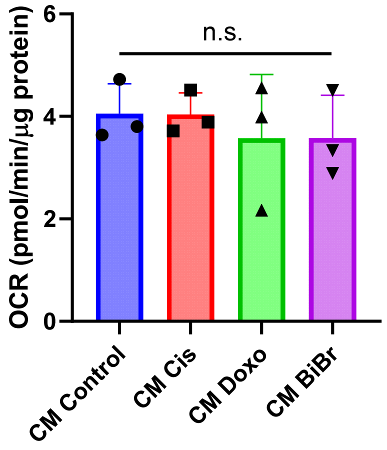

Supplementary Figure S2: Effect of CM from RMF-621 treated with DNA-damaging drugs on ATP-linked respiration of MCF7 cells. Data are shown as the mean ± SD of three independent experiments. n.s.: not significant.

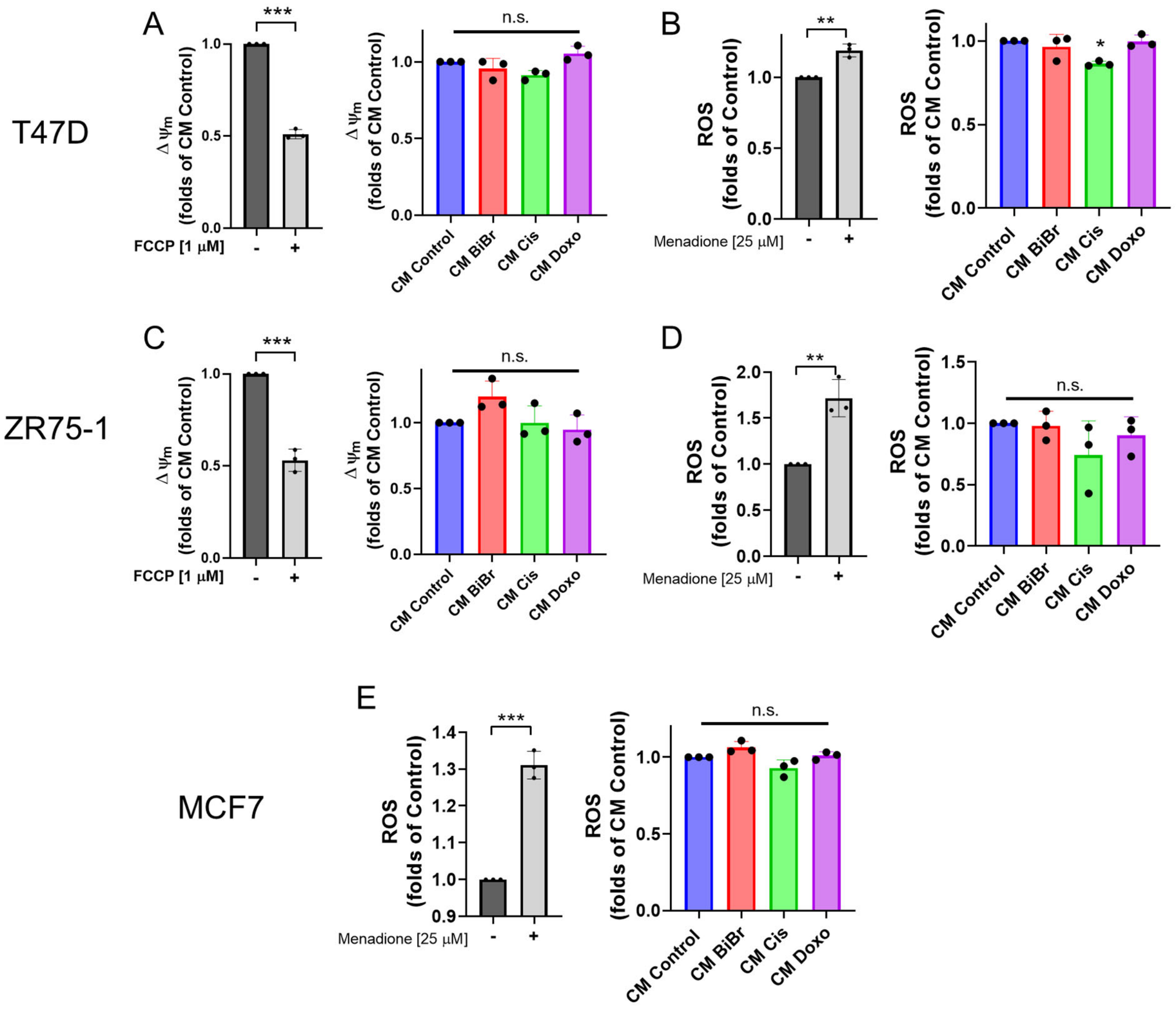

Supplementary Figure S3: Effect of CM from RMF-621 treated with DNA-damaging drugs on mitochondrial membrane potential (Δψm) and ROS levels in low-migrating breast cancer cells. **A, C.** Mitochondrial membrane potential, **B, D, E.** and superoxide production in low-migrating breast cancer cell lines (T47D, ZR75-1, MCF7 cells) exposed to CM from stromal cells for 24 h. The Δψm and ROS levels were determined using TMRM (5 nM) and DHE (5 μM). FCCP (1 μM) and menadione (25 μM) were used as positive controls. Data are shown as the mean ± SD, N=3 independent experiments. *$p < 0.05$, **$p < 0.01$, ***$p < 0.001$, vs. Control (DMSO) or CM Control. n.s.: not significant.

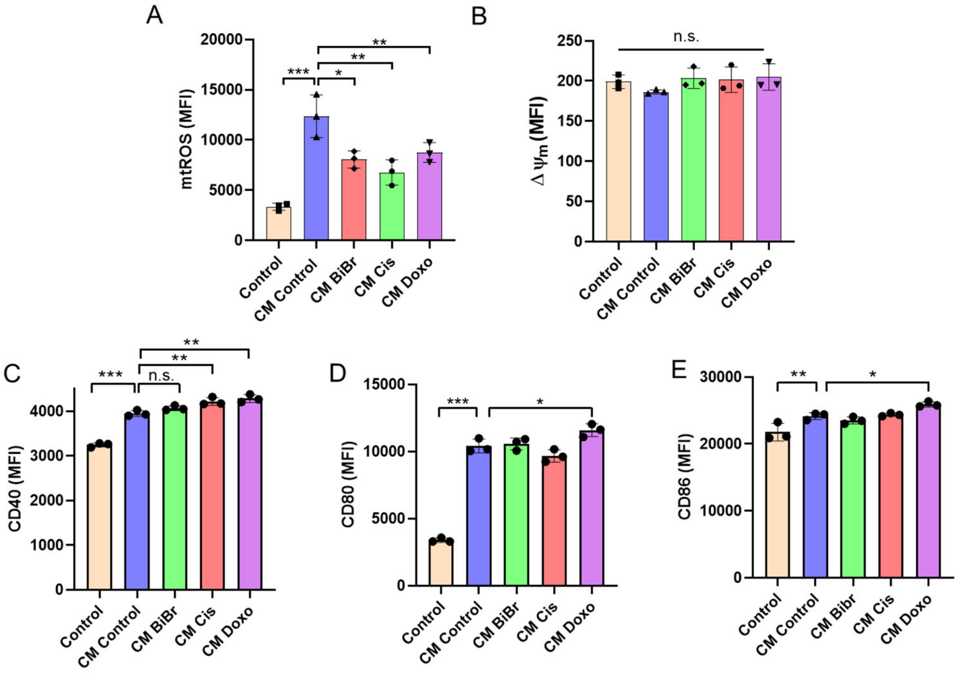

Supplementary Figure S4. Effect of CM from DNA-damaging senescent fibroblasts on bioenergetics and inflammatory membrane markers in THP1-macrophages. THP1-Macrophages were polarized to M1 profile (LPS+IFN 24 h) and then were stimulated with conditional medium from fibroblast exposed to the DNA-damaging drug treatments (24 h). **A.** MitoSOX (mtROS), **B.** Mitochondrial membrane potential, **C.** CD40, **D.** CD80, and **E.** CD86 were measured by flow cytometry. Data are shown as the mean ± SD of three independent experiments. CM= conditioned medium.

Uncropped gels

RMF 621 cells

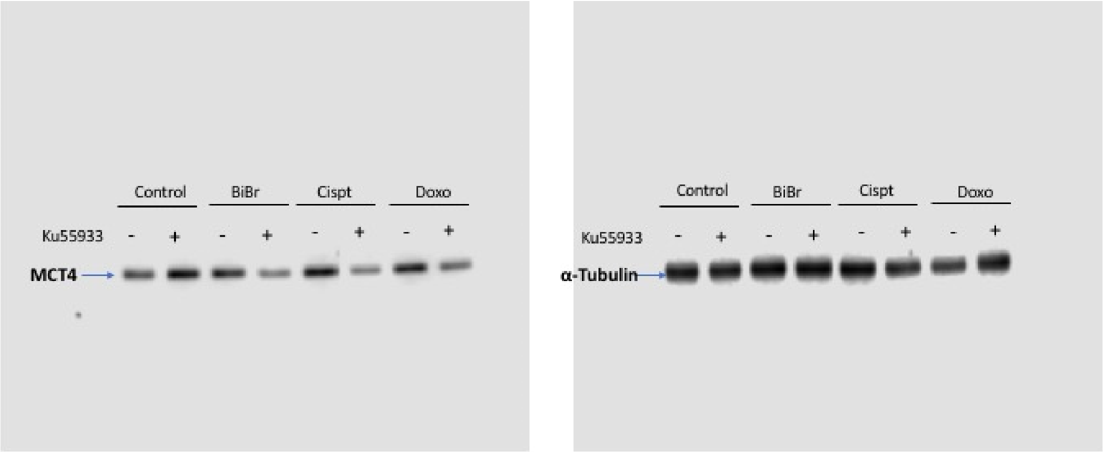

Relative to Supplementary Figure 1C

MCF-7 cells

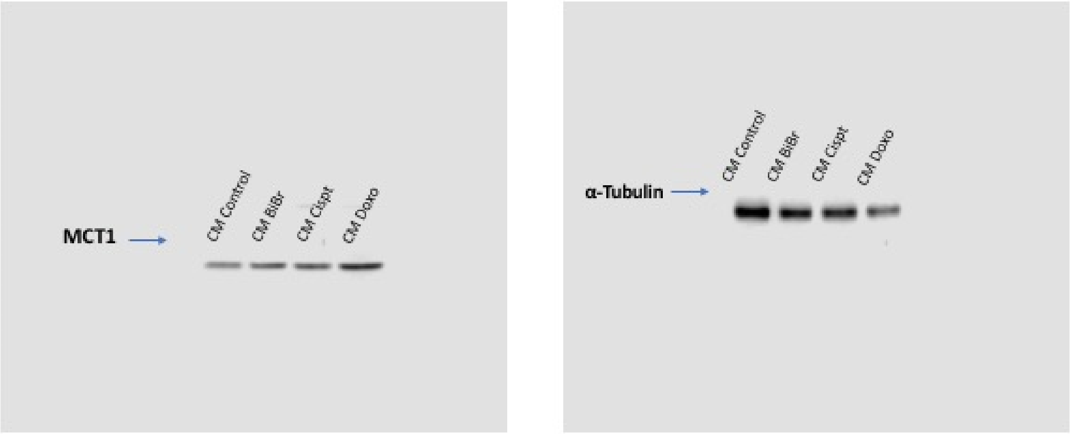

Relative to Fig. 3A

MCF-7 cells

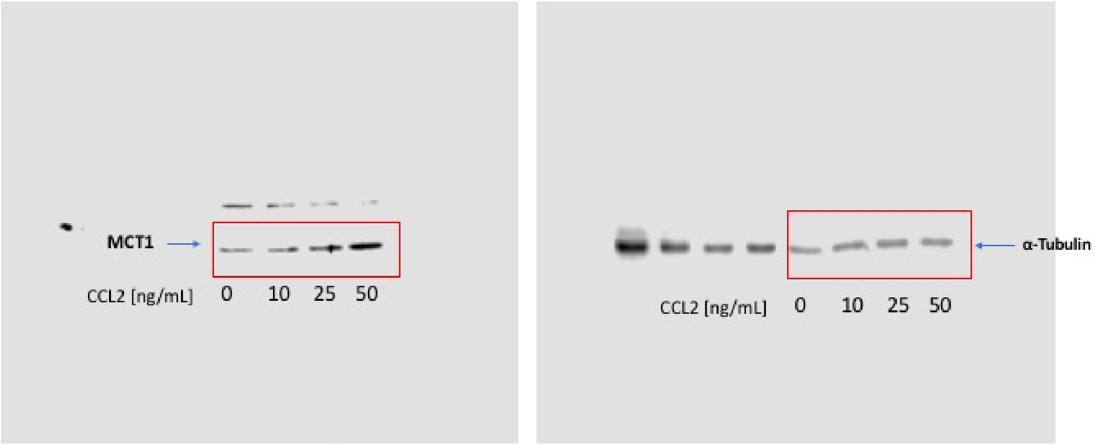

Relative to Fig. 4A
